# A knowledge, attitude and practices assessment of control measures for bovine tuberculosis and brucellosis towards a more effective approach to national control programs in South Africa

**DOI:** 10.1007/s11250-024-04274-7

**Published:** 2025-01-02

**Authors:** Alfred Tlotlo Kgasi, Anita Luise Michel

**Affiliations:** 1 Present Address: Department of Production Animal Studies, Primary Animal Health Care, Faculty of Veterinary Science, Onderstepoort, University of Pretoria, Pretoria, South Africa; 2 Department of Veterinary Tropical Diseases, Faculty of Veterinary Science, Onderstepoort, University of Pretoria, Pretoria, South Africa

**Keywords:** Bovine brucellosis, Bovine tuberculosis, Disease control measures, Knowledge, attitudes and practices, Stakeholder engagement, Wildlife-livestock interface

## Abstract

Bovine brucellosis and bovine tuberculosis are zoonotic diseases with economic and public health importance across the world, especially in developing countries where the diseases are endemic. The diseases are classified as neglected diseases in developing nations with poor resources despite good control measures in some developed countries. The purpose of this study is to assess the knowledge, attitudes and perceptions (KAP) of stakeholders towards control measures for bovine brucellosis (BR) and bovine tuberculosis (bTB) at a livestock-wildlife interface. Stakeholders selected were small-scale farmers and veterinary officials affected by disease control measures in northern KwaZulu Natal in South Africa. A mixed method approach was used (qualitative and quantitative) In the quantitative method, a total of 61 small-scale livestock farmers and 15 veterinary officials working in the livestock-wildlife interface study area, completed a structured questionnaire. In the qualitative method, a focus group discussion was held with each stakeholder group (farmers and veterinary officials) were held using an interview guide that was developed based on common themes/topics emerging from the quantitative method. In terms of farmers, 62.3% and 75.4%, did not know about BR and bTB, respectively. All veterinary officials (100%) knew about BR and bTB. Majority of small-scale farmers (55.7%) and veterinary officials (66.6%) did not believe that the current methods for control of BR and bTB were effective. However, both farmers (55.9%) and veterinary officials (73.3%) believe that the state has capacity to control the diseases provided adequate resources are available, and practical disease control policies are implemented. Farmers (96.5%) and veterinary officials (93.3%) believe that the success of control of BR and/or bTB also depends on both role players working together. In the focus group discussions, reasons provided were, lack of sufficient resources (people and funding) by the government. Communal farming infrastructure was stated as a hindrance to effective disease control. Poor training of farmers and unrestricted animal movement had negative effect on disease control. Factors such as difficulty in isolating/separating test positive from test negative animals (biosecurity) in communal areas, provincialisation of disease control strategies, lack of enforcement, lack of compensation for farmers, were raised by stakeholders. It is recommended that the current structure of the South African veterinary services’ delivery model be revised to enable coherent disease control co-ordination between National Department and Provinces. The current failures to successful control of BR and bTB can be attributed to limited stakeholder engagement and analysis of stakeholder perspectives. To address this, a bottom-up approach that involves inputs from stakeholders on the ground can help enhance the successful implementation of control strategies. The outcome of the study should serve as guide for policy development and implementation for both BR and bTB control measures in South Africa.

## Introduction

Bovine brucellosis (BR) is a highly contagious zoonotic disease that is important in both humans and livestock. The disease is caused by various species of the genus *Brucella*, which tend to infect a specific animal species. *B. abortus* can cause abortion in cattle, and occasionally in sheep and goats (Coetzer et al. 2004). Bovine tuberculosis (bTB) can be described as an infectious disease caused by *Mycobacterium bovis* that affects cattle, other domesticated animals and a wide spectrum of free ranging or captive wildlife species (Office International des Epizooties Terrestrial Manual 2018).

Both BR and bTB have shared characteristics of being zoonotic diseases with economic and public health importance across the world, especially in developing countries where both diseases are endemic (WHO Report 2005; Muma et al. 2013). It is suggested that for greater impact and cost–effectiveness of control measures against zoonotic diseases, it would be advantageous to group the zoonotic diseases together (WHO Report 2005). In Africa, the incidence of both diseases in humans is under-reported due to the non-specificity of the signs of BR and the limited diagnostic capabilities with regards to tuberculosis (Wojno et al. 2016; Ayele et al. 2004; Ducrotoy et al. 2017). Both BR and bTB are chronic diseases by nature and have the ability to persist for several years and often lifelong (Constable et al. 2017; Shitaye et al. 2007; De Vos et al. 2001). The test-and-slaughter approach seems to be a common thread in most effective and successful eradication campaigns for BR and bTB (Constable et al. 2017; More et al. 2015; Chambers et al. 2018).

The current control measures of BR and bTB in South Africa are largely based on approaches recommended by WOAH of test-and-reduction of reservoir hosts, quarantine and depopulation (DALRDD National Brucellosis Control Policy 2020). These approaches may not necessarily be effective and appropriate in the South African context, given the complex socio-economic challenges and integral role of wildlife in the country. Control measures need to be effective, practical and culturally and ethically acceptable to ensure successful control of bTB in South Africa (Arnot and Michel 2020). Recent studies in South Africa have also shown the re-emergence of bTB in commercial cattle production and high variability in prevalence of the disease in the communal cattle farming sector. The effective control of both Br and bTB is a serious challenge in South Africa. Community participation in development of animal and public health programmes is necessary in livestock disease control for a sustainable livestock health policy (Waziri 2020; Muleme et al. 2017).

More than 70% of these resource-poor farmers in South Africa rely on livestock for their livelihoods, and it is therefore important, that their animals are free from disease (Reddy et al. 2016). Veterinary officials, as a stakeholder, are the custodians of animal health and play an important role in providing extension services to farmers and implementation of disease control policies. (Reddy et al. 2016; Caron et al. 2013). Insufficient regulatory and socioeconomic factors and control programs in domestic livestock have been found to play a role in the re-emergence of BR in both livestock and humans in recent years (Scott McVey et al. 2013: 130). The understanding of knowledge, attitudes, and practices of affected stakeholders (small-scale farmers and veterinary officials) towards BR and bTB control measures in livestock, especially at the livestock-wildlife interface, will assist in improving disease control strategies. The wildlife-livestock interface in South Africa allows livestock and wildlife to share space and resources and plays a role in disease transmission (Gomo 2015; Bengis et al. 2002).

## Materials and methods

### Study area

**Fig. 1:**
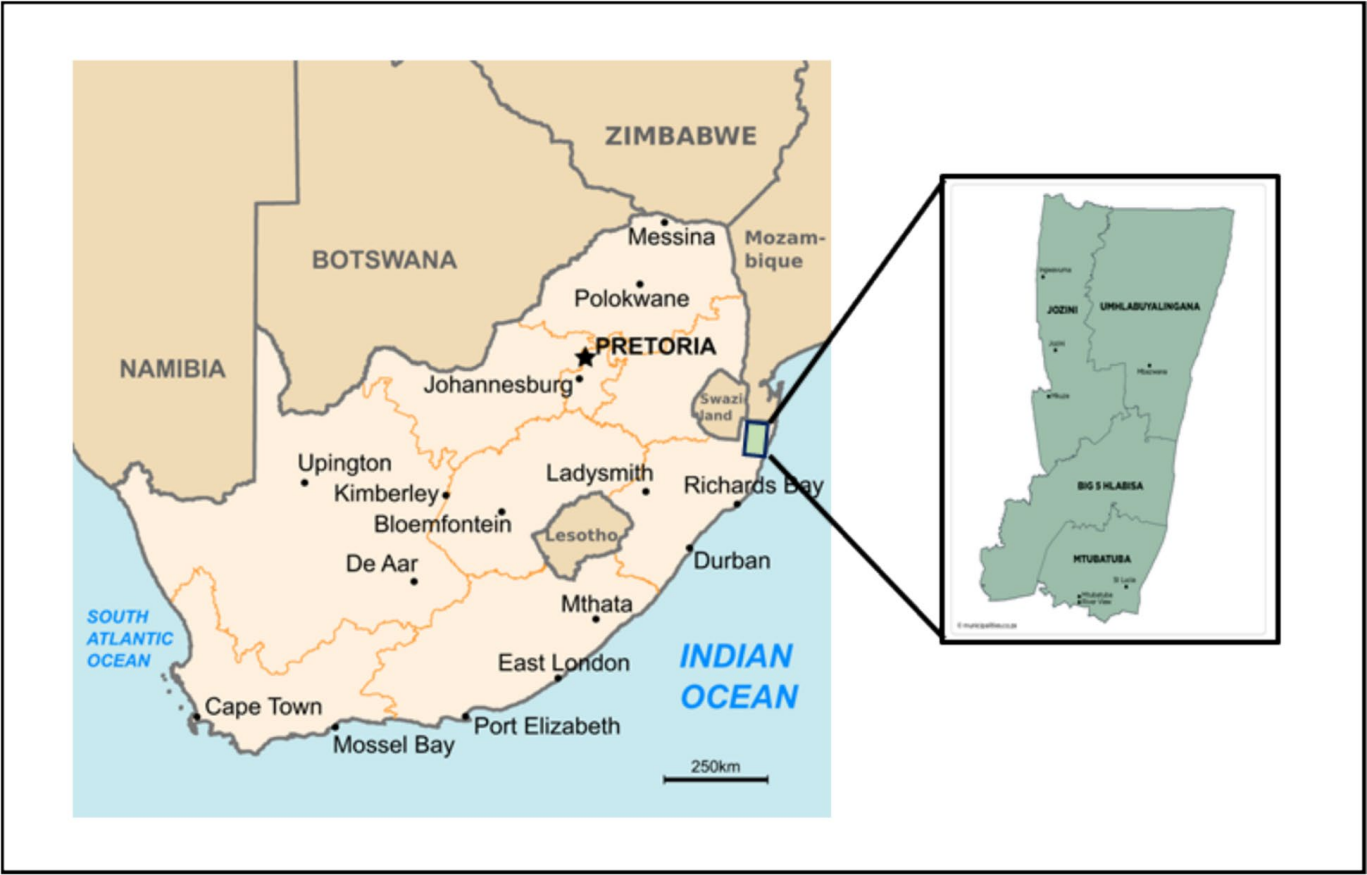
Map of the study area in uMkhanyakude District, northern KwaZulu Natal in South Africa (adapted) (Source-https://freeworldmaps.net)

The study was conducted in the uMkhanyakude District Municipal Area (Big 5 Hlabisa Municipality) in northern Kwazulu-Natal province, in the Republic of South Africa (Fig. 1). The dominant land tenure of the district is communal lands with narrow strips of commercial lands. Over 21% of the district is under proclaimed wildlife conservation with communal livestock farmers interspaced within and/or the borders of the wildlife parks. These livestock owners are farming in communal lands that are at the interface with state and private game reserves such as iSimangaliso Wetlands Park, Mkhuze Game Reserve, Hluhluwe-iMfolozi Park, and Munyawana Game Reserve. State veterinary services are mainly administered by the local Hluhluwe state veterinary office, and also by other neighbouring district state veterinary offices where applicable.

The study area is made of 4 (four) villages namely Nibela, Masakeni, Mpempe and Nkomo at the livestock-wildlife interface in the Big 5 Hlabisa Municipality and uMkhanyakude District in Kwazulu-Natal Province. The estimated cattle population in these villages is 8,000 heads of cattle that are owned by over 400 farmers (Communications, Warren McCall, Control Animal Health Technician 2019). Two groups of stakeholders (respondents) in the area were selected for the purpose of the study. These groups were small-scale livestock farmers (*n* = 61) who practise traditional (communal) farming in 4 (four) rural villages in the study area of Nibela, Masakeni, Mpempe and Nkomo which were previously reported to have had incidences of either bovine brucellosis and/or tuberculosis; and veterinary officials (*n* = 15) based mainly at the Hluhluwe state veterinary office (under uMkhanyakude District Municipal area), and adjacent state veterinary offices (Vryheid, Pongola and Nongoma) responsible for execution of disease control measures at the selected livestock-wildlife interface. Cattle farmers from each of the 4 (four) villages under the jurisdiction of the Hluhluwe district veterinary office were non-randomly selected for the study. The state veterinary officials assisted in inviting farmers from the four villages to participate in the study. BR and bTB have been diagnosed in all the villages. All the veterinary officials who were at that time of the study under the employment of the local Hluhluwe district state vet office, were selected for the study.

### Data collection

#### Stakeholder questionnaire completion and focus group discussions

In the quantitative method, structured questionnaires were completed by both small-scale farmers (*n* = 61) and local veterinary officials (*n* = 15) as shown in Table 3. The questionnaires included closed and open-ended questions; and due to the possibility of limited literacy skills within some small-scale farmers, the questionnaires were completed face-to-face. Questionnaire completion sessions were held with the farmers from each of the four selected villages i.e. Nibela, Masakeni, Mpempe and Nkomo villages. In each session, the facilitator, translator and a local animal health technician assisted farmers in completing the questionnaire. The questionnaires were also available in the local language of *isiZulu.* Upon completion, the questionnaires were translated to English and coded for analysis. The veterinary officials were sent questionnaires electronically and once completed, were sent back to the researcher. There was an average of 15 farmers per village i.e. Nibela (11), Masakeni (17), Mpempe (15) and Nkomo (18). For data analysis the responses from the participating farmers in all the 4 villages were combined as the traditional farming practices, socio-economic status and climatic conditions in the respective villages are similar.

A scale was developed to classify the levels of awareness/knowledge of disease to ensure a uniform interpretation of data. The scale was as follows; 0 – 39% was regarded as low levels of awareness/knowledge; 40 – 59% regarded as medium level of awareness/knowledge, and 60—100% regarded as high levels of awareness/knowledge.

In the qualitative method, a focus group discussion was held with each stakeholder group. An interview guide developed based on the summarised themes/topics (disease control measures) emerging from the quantitative method analysis, was used. These themes were (a) awareness and disease transmission (b) vaccination (c) testing and surveillance (d) movement control (e) culling and compensation (f) knowledge transfer (g) implementation capacity, as summarised in Table 1 below.

**Table 1:** Measures for control for BR and bTB

	Areas of Focus in Disease Control Measures
1)	Testing (epidemiological surveillance)
2)	Vaccination (except bTB)
3)	Movement control
4)	Biosecurity
5)	Prevention of livestock-wildlife contact
6)	Test-and-slaughter strategy
7)	Promotion of food safety
8)	Education/Knowledge transfer

With regards to farmers, the focus group, consisted collectively of fifteen (15) farmers selected through volunteer participation per village (Nibela, Masakeni, Mpempe and Nkomo villages), All 15 participants in the focus group session had participated and completed the original questionnaire. The venue was well-ventilated with good lighting and participants were seated in chairs arranged in a semi-circle configuration. The discussion session was conducted for two hours with a break of 30 min within the session. Light refreshments were served during the break. The respondents were allowed to communicate in their mother-tongue (isiZulu). An experienced translator who has done previous farmer trainings and who is conversant with the local language facilitated the discussions. The translator was also given training on the use of the questionnaire. Responses were recorded via an audio device and hand notes also be typed and/or written during the proceedings of the interview. With regards to veterinary officials, the focus group consisted of nine (9) respondents. The discussion session was also about 2h 15 min with a break of 25 min during the session. Light refreshments were served during the break. The room was well ventilated with an ambient temperature. The discussions were held mainly in English but provision was made for some participants who responded in the local language (isiZulu). Responses were recorded via an audio device and hand notes were also typed and/or written during the proceedings of the interview.s. One focus group discussion (FGDs) with each stakeholder group.

### Data management and analysis

In the quantitative method, the data that was collected through a structured questionnaire, was analysed using a SPSS statistical package. The proportions were calculated with the 95% confidence intervals using Mid-P exact and the association between categorical variables were calculated using the Pearson's chi-square test of hypothesis with values < 0.05 classified as significant. Categorical variables, including nominal and ordinal variables were described by tabulating their frequencies. If two values were associated, the probability of one will depend on the probability of the other.

In the qualitative method, the data was mainly deductive but allowed a part open for inductive approach when identifying topics and common themes emerging from the quantitative method (questionnaire). The data was transcribed and coded using phrases that represent important and common themes. Thematic content analysis was utilized through identification of common issues that recurred and creation of themes out of the summarised views collected.

## Results

The results reflect the findings on the quantitative and thereafter qualitative research method findings. The qualitative analysis was mainly deductive and the topics/themes were developed from the quantitative method findings. The focus group discussion, therefore, expanded and gave deeper and useful insights into the questionnaires. Quantitative and qualitative results are presented concurrently.

### Questionnaire and focus group discussions

#### Demographics

The small-scale farmers, consisted of males (*n* = 57) and females (*n* = 4). Majority of the respondents were between the ages of 35 – 55 years (49.2%), followed by over 55 years (44.3%), and lastly 18 – 25 years (6.6%) as shown in Table 2. They had varying levels of educational backgrounds, majority of the respondents only had primary level education (39.3%), high school (31.1%), university/college (9.8%) and no education (19.7%). Most of the respondents were unemployed (62.3%), employed (11.5%), self-employed (3.3%), and pensioners (23%). Most of the respondents (93.4%) graze their animals on communal pastures, 4.9% on private land, and 1.6% on trust land. Their herd sizes are 11 – 50 cattle (56.7%), 10 or less (26.7%) and above 50 (16.7%).

**Table 2:** Demographics and farming details for small-scale farmer respondents

Characteristic	Category	Proportion (%)
Small-scale farmers (*n* = 61)		
Gender	Male	93.4
	Female	6.6
Age	18 – 25 years	6,6
	35 – 55 years	49,2
	> 55 years	44,3
Education	No education	19.7
	Primary school	39.3
	High school	31.1
	Tertiary education	9.8
Employment status	Unemployed	62.3
	Self-employed	3.3
	Employed	11.5
	Pensioner	23.0
Farming details		
Herd size	≤ 10 cattle	26,7
	11 – 50 cattle	56.7
	> 50 cattle	16.7
Grazing	Private land	4.9
	Communal land	93.4
	Trust land	1.6

The veterinary officials (*n* = 15) were composed of animal health technicians (46.7%), senior animal health technicians (20%), and veterinarians (33.3%) as shown in Table 3. All the respondents (100%) had either an animal health diploma/degree or a degree in veterinary science. Majority of the respondents (66.7%) had more than ten years’ experience in their jobs as veterinary officials. All respondents (100%) work in areas where cattle are one of the main livestock species of importance.

**Table 3:** Demographics of veterinary officials

Characteristic	Category	Proportion (%)
Veterinary officials (*n* = 15)		
Gender	Male	80
	Female	20
Age	18 – 25 years	13.3
	35 – 55 years	80
	> 55 years	6.7
Education	High school	0.0
	Tertiary education	100.0
Position at work	Animal Health Technician	46.7
	Senior Animal Health Technician	20.0
	Veterinarian	26.7
Experience at work	2 years or less	6.7
	3 – 5 years	6.7
	6 – 10 years	20.0
	> 10 years	66.7

The following findings were obtained for the seven themes and are summarized in Table 4.

- Awareness of the diseases and their control measures

**Table 4:** Summary of findings relating to knowledge, attitudes and practices regarding BR and bTB among small-scale farmers and veterinary officials

Disease control aspect	Farmers						Veterinary Officials					
	BR			bTB			BR			bTB		
	Yes	No		Yes	No		Yes	No		Yes	No	
Do you know about the disease	62.3	37.7		75.4	24.6		100	0		100	0	
	Agree	Neutral	Disagree	Agree	Neutral	Disagree	Agree	Neutral	Disagree	Agree	Neutral	Disagree
The disease can be completely eradicated in your area	55.9	28.8	15.3	63.9	31.1	3.3	73.3	6.7	20.0	20.0	6.7	43.3
All cattle should be tested for the disease	92.9	5.3	1.8	98.3	1.6	0.0	86.7	6.7	6.7	13.4	40.0	46.7
Vaccination is a good method for controlling the disease/All cattle must be vaccinated	98.2	0.0	1.8	91.8	6.6	2.6	60.0	6.7	33.3	100.0	0.0	0.0
Animals with the disease must not be allowed to leave the farm/Movement must be restricted on + ve animals	82.5	10.5	7.0	93.4	0.0	6.6	100.0	0.0	0.0	100.0	0.0	0.0
I would not buy/or recommend buying cattle from a diseased herd	75.4	15.8	8.8	83.7	4.9	11.5	86.7	0.0	13.3	100.0	0.0	0.0
Cattle with the disease must be killed/culled to stop the disease	33.3	38.6	28.1	31.1	31.7	37.7	100.0	0.0	0.0	100.0	0.0	0.0
Compensation mechanisms for culling of infected animals are adequate	n/a	n/a	n/a	n/a	n/a	n/a	26.7	0.0	73.3	6.7	13.3	80.0
Separation of cattle from wildlife can stop the disease	75.4	19.3	5.3	82.0	13.1	4.9	66.7	26.7	6.7	93.3	0.0	6.7
All abortions should be reported to veterinarian and animal health technician for investigation	94.7	3.5	1.8	98.3	0.0	1.6	66.7	26.7	6.7	66.7	26.7	6.7
The farmer, animal health technician and veterinarian must work together to control the disease	96.5	3.5	0.0	98.4	0.0	1.6	100.0	0.0	0.0	100.0	0.0	0.0
The current methods of controlling the disease are effective	46.4	35.7	17.9	55.7	29.5	14.8	13.3	26.7	60.0	6.7	26.7	66.3
Practices												
Items	Yes	No		Yes	No		Yes	No		Yes	No	
Have (your) cattle ever been tested for the disease?/Do you test cattle for the disease	19.7	80.3		57.4	42.6		100	0		73.3	26.7	
Are the cattle you buy tested for the disease?	18.0	82.0		24.6	75.4		n/a	n/a		n/a	n/a	
Have your cattle ever been vaccinated against the disease/Have you vaccinated cattle against the disease	31.1	68.9		31.1	68.9		100	0.0		0.00	100.0	
Do cattle get vaccinated against the disease in your area	n/a	n/a		n/a	n/a		100	0.0		0.00	100.0	
Do your cattle ever come into contact with wild animals?	50.8	49.2		50.8	49.2		100.0	0.0		100.0	0.00	
Do your animals ever graze inside the game reserve?	39.3%	60.7%		39.3%	60.7%							
Are there proper fences separating wildlife from your cattle?	45.9	54.1		45.9	54.1		96.6	13.3		96.6	13.3	
Did you buy or receive animals that were not tested, from another area during the past year?	16.4	83.6		16.4	83.6		n/a	n/a		n/a	n/a	
Do farmers sometimes buy animals that were not tested, from another area?	n/a	n/a		n/a	n/a		86.7	13.3		86.7	13.3	
Have your farm been closed up (quarantined) due to either brucellosis or tuberculosis?	14.8	85.2		14.8	85.2		73.3	26.7		53.3	46	
Have you ever had (your) cattle killed by the government due to infection with either brucellosis or tuberculosis?	8.2	91.8		8.2	91.8		60	40		20	80	

The quantitative questionnaire assessed the knowledge of respondents about the diseases in a two-stage process. If they responded “Yes” to whether they knew BR or bTB, they were then given several random related and unrelated signs of disease to choose from. This helped the researcher validate if they knew the actual signs of the specific disease. In terms of BR, the majority of the farmers (63.3%) stated that they knew about a disease called brucellosis (also called “abortion disease”) as shown in Table 4. Correspondingly, the majority of respondents (75.4%) stated that they also knew about a disease called tuberculosis (called ‘TB’) and associated signs. In the FGDs, some respondents correctly identified the clinical signs of bTB, particularly relating to debilitation, coughing, weight loss in animals, and characteristic lesions on post mortem. With regards to BR, some participants confirmed that they heard about the disease called brucellosis in cattle; and the disease was called, *“isifo sokuphunza*” in the local language (meaning “*abortion disease”*), although some participants mentioned that they had not heard of the disease before.

*“I heard one of the animal health technicians, talking about it.” (P4, May 2021).*

*“As far as I know, apparently this ‘abortion disease’ of cattle can also affect people. So, it is a dangerous because of that. It can cause miscarriage in pregnant women. Someone told me that it is a disease that causes abortions. I don’t know how this disease causes abortions.” (Farmer 7, May 2021)*

*“People can get the disease from drinking milk from a sick cow. It’s even worse that if a cow has aborted, the farmer starts milking the cow and using the milk. And that’s where I think the biggest danger is.” (Farmer 8, May 2021)*

The farmers knew there were situations where an animal looked healthy but when tested by the veterinary officials, were found to have the disease. There was a strong view that the disease was relatively new in the area and was first seen in the past 5 – 10 years.

*“I know about the disease. From what I know, it makes cattle very sick and they also cough a lot. If you cut the animals open, inside their body cavities, you find small nodules that look almost like river sand”. I know about the disease.” (Farmer 1. May 2021)*

The veterinary officials (100%) knew about BR and bTB and knew that both were controlled diseases in the country. They were able to correctly mention abortions as a major sign for BR; and identified coughing and debilitation as the most common signs for bTB. In the FGDs, some of the participants demonstrated good knowledge of both diseases, the modes of transmission and control principles.

*“Abortions are also not caused by brucellosis. So, if the farmers report aborts, the AHT can also investigate and teach farmers about other causes of abortions e.g. poor feeding, poor condition, etc.” (Official11, May 2021)*

During the FGDs, the officials demonstrated good knowledge of the different methods of controlling BR and bTB, the similarities in the control methods.

*“Test and slaughter is an effective method tuberculosis control” (Official 2, May 2021)*

*“Quarantine and movement restriction is an important element of tuberculosis control” Official 8, May 2021)*

- Vaccination

In terms of BR, a significant majority of farmers (98.2%) agreed that vaccination is a good method for controlling BR. In terms of bTB, most of the farmers (91.8%) also believe that vaccination would be a good method of controlling bTB. They believe that BR and bTB can be eradicated if the government can provide them with vaccines or medicines. They mentioned that the government is not providing them with the vaccines. Most of the respondents (68.9%) stated that their cattle have never been vaccinated against BR. During the FGD, farmers mentioned that the government used to do vaccination in the past; however, due to budgetary constraints it was happening less often. Farmers were not buying their own vaccines. Therefore, vaccine availability from the state influences the vaccination coverage in the study area, with only 31.1% of farmers having had a chance to get their animals vaccinated by the state at some stage in the past years. These animals that were vaccinated by the state, were those animals that were by coincidence at the diptank on the days when vaccines were available and administered on those days.

*“I think the disease can be eradicated if the government can provide us with the vaccines to protect our animals from getting the disease.” (Farmer 8, May 2021)*

*“I think vaccination helps prevent my animals from getting the disease. It is better than having to treat sick animals. (Farmer 9, May 2021)*

In terms of bTB, the officials indicated that there was a need to more seriously consider vaccination as a tool for control of bTB in future. All veterinary officials (100%) indicated that cattle do get vaccinated against BR in their areas of work. During the FGDs, the officials mentioned a lack of willingness amongst farmers to bring animals for vaccination. The officials believe this could partly be attributed to concerns related to the safety of the S-19 brucellosis vaccine in pregnant animals.

*“One of the challenges is that because some animals may be pregnant when brought to the diptank, we always ask the farmer whether their animals are pregnant or not. So, farmers then become worried that the vaccine may negatively affect the pregnancy status of the animals …”. (Official11, May 2021)*

*“Maybe it's time we also open a conversation about possible vaccinations against TB in cattle or buffaloes. I know it that National Department there may not want to hear about. I know in Spain that they have had some success with vaccination in wild animals. Therefore, perhaps vaccination can assist in potentially eradicating the disease. The current experimental vaccines could perhaps provide some hope.” (Official 4, May 2021)*

- Testing and surveillance

In terms of BR, the majority of the farmers (80.3%) indicated that their cattle have never been tested for BR. In terms of bTB, 42.6% of the farmers indicated that their cattle also have never been tested for bTB. However, the farmers (75.4%) indicated that they would not buy cattle from a herd known to have BR and 92.9% of respondents agreed that all cattle must be tested for BR. During the FGDs, most farmers mentioned that they felt it was necessary for their cattle to be tested for BR and bTB. The farmers were of the view that all farmers must be required to have their animals tested by bringing animals to the diptank for testing.

*“I think it is important to test animals so that we can know which ones are sick and which one are healthy. That’s important for me as a cattle owner.” (Farmer 2, May 2021)*

*“The problem is we don’t get given the feedback on the tests once they have been done. I think after doing research or testing our animals, the government must come back, gather us together and give us feedback on the test results and the way forward. Sometimes they do the test and 6 months or even a year passes without us being told of what the outcome is or solution should be.” (Farmer 1, May 2021)*

Majority of farmers (82.9%) agreed that testing of cattle for BR can lead to eradication of the disease. Similarly, most veterinary officials (86.7%) also agreed that cattle must be tested for BR. The veterinary officials stated that one of the challenges with testing was that it is voluntary (not compulsory). The officials also reported that they feel that testing is done simply ‘to tick boxes’ as there was no consequence or further action if animals test positive. It was clear that the participants were frustrated and felt powerless about inability to take steps and enforce the culling or removal of animals.

*“So, in order to eradicate the disease, there has to be enforcement or law that says that all animal owners must bring their cattle to the diptank for treatment in order to be able to eradicate the disease.” (Farmer 10, May 2021).*

*“The current scheme is geared only to commercial farmers […] It did not focus on all farmers, including communal farmers.” (Official8, May 2021)*

*“…. But in truth, the quarantine is only on a piece of paper, and nothing happens in practice. But I say that it's under quarantine for what it's worth. It's just because it's a piece of paper in terms of practical control. There was an area where animals tested positive. There was nothing happening that we could do because they say there is no money for vaccination. The Government vet was involved, but all he could was just to put the place “under quarantine” in terms of the paper work.” (Official2, May 2021)*

During the FGDs, the veterinary officials mentioned the cost and supply of equipment was one of the obstacles to effective testing of animals. Most participants (68%) mentioned that the testing for bTB was labour intensive and often farmers did not comply with bringing back the animals to the dip tank for reading of the skin tests which was a problem. They said more testing is focused on commercial farms as some of those farmers are exporting. The cost of bTB was mentioned to be prohibitive for extensive testing. The participants mentioned that there were challenges with availability and standards of training in reading and interpretation of tests.

*“I think the one big thing about TB control is that is that the equipment is expensive. So, if you're going to do the comparative test, it's damn expensive.” (Official 2, May 2021)*

*“The biggest challenge is to get the farmers to complying with bringing the animals for skin test reading after 72 hours. There is very low compliance and I’m not sure what they are not interested in bringing them back. Maybe they feel it is too much work.” (Official 6, May 2021)*

*“Doing the interpretation of a skin test is another massive challenge…...It's easy if everything's perfect and no swelling, but the moment you start getting skin reactions, that becomes a challenge of interpretation…whether it is positive or negative?” (Official 2, May 2021)*

- Movement Control

The farmers (54.1%) indicated that there are no proper fences separating wildlife from their cattle, and some of their animals graze inside the game reserves. The farmers (50.8%) stated that their cattle have come into contact with wild animals. In the FGDs, they mentioned that there is a lot of movement of animals in the area due to purchase and movement of animals from different areas. The veterinary officials also mentioned there was no effective movement control in the province. The officials felt that cattle should be treated the same as buffaloes when it comes to disease control.

*“I think there’s a lot of movement of animals because people buy and sell animals all over the place (Farmer 11, May 2021)*

*“…I think movement of cattle can contribute in the spread of the disease.” (Farmer 4, May 2021).*

*“We do not have any control over the movement of animals. Most of the time we are not even aware that animals are moving or not.” (Official 4, May 2021)*

*“I think through controlling movement of animals, that’s where we can control the disease.” (Official 6, May 2021)*

*“According to me, there’s no logic in dealing with the two species, cattle and buffaloes, differently. If you impose measures on one species, then you need to impose the same measures on the other. Because what is really the point to differentiate them? (Official 2, May 2021).*

- Culling and compensation

Only 33.3% and 31.1% of farmers agreed that cattle with BR and bTB, respectively, must be culled to stop the disease. In the FGDs, some farmers seemed not to prefer that all cattle be killed when infected with BR and/or bTB. However, all farmers confirmed that they do not trust that the government will compensate them when their animals get culled for either BR or bTB. The farmers indicated they had no problem with culling the animals that are sick, as long as they were compensated. However, the veterinary officials stated that they had cases where animals tested positive, and nothing could be done about the situation. They mentioned that even though the BR control policy mandates the removal and culling of infected animals Government did not have the capacity to enforce that. This situation, the participants mentioned, is very frustrating.

*“We love our animals, and again we don’t trust that government will compensate us when we they cull our animals.” (Farmer 3, May 2021)*

*“I do not have a problem with the Government taking or destroying the animals, but the Government must pay me back for my animals. The challenge is that the Government tells us that they do not have money to do compensation. The whole thing falls flat because there’s no exchange for the animals that they want to take.” (Farmer 1, May 2021)*

*“I don’t think culling can work, if it doesn’t go together with compensation. Any culling has to go together with compensation, for it to be successful.” (Official 10, May 2021)*

*“We don't even know the procedure of how it works? We haven’t even seen it. This issue does not even get discussed in management meetings. It is a taboo topic. …. there is no SOP on compensation. You are not even allowed to talk about compensation to the farmer. Because there’s nothing that will be done to compensate the farmer. (Official 4, May 2021)*

- Knowledge Transfer

The farmers (96.5%) believe that the farmers, animal health technicians and veterinarians must work together to control BR or bTB which was echoed by 93.3% of the veterinary officials. The farmers mentioned that in instances where the government cannot provide medicines, they are willing to buy the medicines provided they are advised on what to buy. The farmers mentioned that they are not getting adequate training and advice from the government officials and therefore the government should educate them about the disease and its prevention.

*“I think whilst we are waiting for the government to provide medicines, at least in the meantime, they must give us a list of medicines that we can buy ourselves in order to help ourselves. The problem is that the government is not telling us or giving us the list of medicines that we need to get. We do not know what to buy. Such knowledge will empower us get the medicines ourselves.” (Farmer 10, May 2021)*

*“The government has the solutions to many of these diseases and the farmer has to work with them to get the answers.” (Farmer 4, may 2021)*

One of the challenges raised by veterinary officials (78.0%), was that farmers did not routinely report diseases related to sub-clinical conditions where the signs of disease are not overt or obvious to the farmer. Therefore, farmers tend to focus only on diseases that are more visible and dramatic, and report only those. They felt there is a need for more scientific research and expansion of knowledge *on the dynamics of the disease in areas as susceptible species, transmission and control methods.*

*“I think it's because visually when you look at the animal, it doesn’t look sick and the animal is not dying. So, the moment animals start dying, then the farmers will report. But that animals walking around being infected, but he doesn't see the disease see physically, there's something wrong with this animal. So, it doesn't bother him.” (Official 3, May 2021)*

*“It can be eradicated if there’s more scientific knowledge in terms of on susceptible species, transmission methods, and morbidities. I think currently there's not enough knowledge to be utilized in the effective control of TB.” (Official 5, May 2021)*

- Implementation Capacity

The farmers (46.4%) do not believe that the current methods for control of BR are effective, 35.7% are not sure, and only 17.9% agree the methods are effective. They believe that the disease has been in the area for long and that most farmers do not have information about of the disease. However, most of the respondents (55.9%) agreed that BR can be eradicated in their farming areas, 15.3% did not agree and 28.8% were neutral. Reasons provided were that participants trusted the expertise of the veterinary officials and the capacity of government to assist them. During the FGDs, the farmers mentioned that the government is not committed in assisting them with disease and that communal farming set-up does not enable effective disease control.

*“[…] There are no camps or no fences in communal areas. We have raised the issues with government for many times, by they don’t do anything about it. This issue is really making me very angry (sighs). Where is our hope?” (Farmer 1, May 2021)*

*“I think the problem is that the Government does not have enough personnel to do the work. The officials are not able to cover all areas. They try to cover everyone, but it is just impossible (Farmer 1, May 2021)*

*“I think the biggest problem is when the owner does not take responsibility. When the animal has for example tested positive for the disease, because the animal still looks healthy the farmer still keeps it and doesn’t get rid of it or kill it. …. He sees the cow looks good despite being told that it is positive and he decides to keep the animal (Farmer 14, May 2021)*

In terms of BR, most of the veterinary officials (66.6%) indicated that they do not think that the current BR strategy is effective, 26.7% unsure and only 6.7% think it is effective. Most of them (73.3%) agreed that BR can be eradicated in their areas of work, 20% did not agree and 6.7% were neutral. Those that agreed it can be eradicated based their reasons on reported successes in other countries, current expertise within Government, possible improved collaboration between farmers and veterinary officials, and possible funding by Government. Reasons for a belief that it cannot be eradicated were stated as difficulty isolating animals in communal grazing, current ineffective control strategies, lack of compensation for farmers, and lack of resources and personnel.

*The Government also has to provide the resources to enable the programme to happen. This will include, in the control strategy, increasing abattoirs where infected animals can be slaughtered. Maybe providing abattoirs with PPE’s etc. There are costs in culling animals. The Government will have to provide subsidy for transportation of animals to abattoirs. I think the politicians have not seen the importance of controlling the disease.” (Official 4, May 2021)*

The officials reported challenges with the implementation of disease control strategy due to each province doing its own thing. The participants indicated that the whole BR control strategy was not cohesive due to provincialization of implementation of the strategy. They explained that there was no co-ordination at inter-provincial level and also between provinces and the National Department. They mentioned that this challenge was leading to failure in the successful execution of BR control strategy. The veterinary officials in as much as they want to enforce the rules, they mentioned that they are not getting the necessary support to enable them me to do the work, without potentially running into trouble.

*“Still on the Government side, the main problem is the provincialization of veterinary services. Each province does its own thing depending on its political heads or provincial administration structure. There’s no specific coordination. So, this province decides I'm not running with that and the other province decides on something else.” (Official 2, May 2021)*

*“One thing that I've noticed is that with democracy coming in, people have more power and can disregard what you are telling them, even if you mention that by law that this is not supposed to happen. The farmers will just tell you straight that what you are saying won't happen. And therefore, we feel powerless, as there isn't much we can do in terms of the law enforcement side of our jobs. The law enforcement as part of our job in fact does not exist. Even the police won’t accompany you if you want to enforce something.” (Official 4, May 2021)*

## Discussion

This study was conducted with the aim to determine the knowledge, attitudes and practices of stakeholders towards disease control measures related to BR and bTB in the KwaZulu-Natal Province of South Africa. Successful disease control measures in general, are inextricably linked with what the affected stakeholders know, perceive and practise. Farmers in the study area had an expectation that the government should assist them in controlling these diseases. The risk in terms of the Theory of Planned Behaviour (TPB) is that through farmers’ expectations that the government had to assist them, they would not necessarily take active steps to control the disease themselves. TPB has shown in the study how attitudes can influence practices affecting disease control. The awareness of bTB was high (75.4%) amongst the participants and can be partially attributed to other studies including awareness campaigns conducted in the area (Sichewo et al. 2017; Sichewo et al. 2020a, b). A study in Zambia also confirmed that cattle owners with good knowledge of bTB were those with previous exposure to bTB control (Munyeme et al. 2010)*.* The high levels of awareness of both BR and bTB by farmers can thus be a useful basis for veterinary control interventions.

The perception by farmers that vaccination is the best method to control BR (and bTB as well) and that the government has the knowledge and expertise to control the diseases is a significant finding. This could imply that farmers are fully dependent on government to provide the solutions to the disease problems. The perception of high vaccination rates of between 60 – 70% as reported by veterinary officials was sharply contrasted by the perceived vaccination rate of 31.1% mentioned by the farmers. This could be attributed to a lack of communication between officials and farmers when vaccinations are conducted by officials at the diptank. Both farmers and veterinary officials supported the compulsory testing of livestock critical to enhancing the control programme of both BR and bTB. However, testing of animals without due actionable consequences has no epidemiological benefit and creates frustration amongst officials, and distrust from farmers.

Gilbert et al. (2005) reported cattle movement to be an important and consistent risk factor in the transmission of bTB and often leading to outbreaks. There is no effective control of movement of livestock in the study area due to cattle moving freely between grazing areas, into reserves, sale of animals and possible use of the cattle for traditional practices such as *lobola* (similiar to dowry) in rural parts of KZN province. This finding was corroborated by both the farmers and veterinary officials. It was also determined in a study by Sichewo et al. (2020a) that the free movement of cattle in the communal farming areas into game reserves together with sharing of pastures and watering points in the same study area were significant risk factors for bTB in cattle herds. Therefore, restriction of movement and separation of wildlife and livestock are critical measures in the control both BR and bTB in the study area and elsewhere (Cowie et al. 2015; Munyeme et al. 2010; Caron et al. 2013).

Culling and compensation was a very concerning and emotive issue within both stakeholder groups of farmers and veterinary officials. There was an acknowledgement by the farmers and veterinary officials that culling can help reduce or stop the disease, however such removal of animals should be associated with financial compensation. This demand is not unique to South Africa and practised in many countries (Cowie et al. 2015). It has been shown that conventional control measures of disease control such as test-and-slaughter are difficult to implement due to challenges with resources (finance), logistical and political factors (Kilpatrick et al. 2009; Arnot and Michel 2020). The fact that farmers are not willing to have their animals culled if there is no compensation and further do not trust that the government will compensate them, poses a problem. This is aggravated by the uncertainty among the veterinary officials as to the conditions for compensation of cattle farmers for removal of infected cattle.

This study identified gaps and inadequacies in knowledge transfer from the veterinary officials to farmers whereby this, together with dissemination of information on controlled diseases has been flagged as important in order to successfully manage the spread of animal diseases such as brucellosis and bTB in rural communities (Ndengu et al. 2017; Khan and Zahoor 2018). Despite this shortcoming the farmers trust that veterinary officials have knowledge and expertise on the diseases. The knowledge gap among farmers about the diseases provides an opportunity for the veterinary officials to provide farmers with relevant training. Education and awareness campaigns by government on the control and prevention of BR can yield positive results as demonstrated in some countries (Awwad et al. 2017). More scientific research and expansion of knowledge on the dynamics of both BR and bTB in areas such as susceptible species, transmission and control methods*,* was reported to be necessary.

Both the farmers and veterinary officials did not believe that current methods for control of BR and bTB were effective since the diseases have been present in their area for at least several years and have still not been eradicated. Reasons for the ineffectiveness/failure of the government strategy were associated with difficulty isolating animals in communal lands, lack of compensation for farmers, operational structure of disease control, lack of knowledge transfer, inadequate vaccination, and lack of resources and under-staffing. These factors are indicative of the government’s failure in controlling both diseases in the study area.

The fragmentation and provincialization of veterinary services was revealed as a major hindrance in effective implementation of BR and bTB strategy. This challenge was also reported by Bruckner (2014) that even though National Veterinary Services retained national legislative mandate for animal disease control, provincialisation resulted often in dissonance between provincial heads of veterinary services and the National Government. There is a sense of desperation amongst farmers as they feel the government is no longer committed to assisting them with the control of BR and other animal diseases.

Although the stakeholder group of farmers represented largely livestock owners whose herds were affected by BR or bTB and their opinions and perspectives may have been biased by personal experiences or those of neighbours, their feedback and inputs concerning disease control strategies are invaluable to the successful implementation. The study was based solely in the opinions of farmers and officials (most of them are not veterinarians). More data or studies are needed to verify the findings in other areas. Data on prevalence and occurrence of the diseases along the time, number and distribution of the tests, figures of vaccination among other features and data should in future be added to the opinion of stakeholders.

## Conclusion

The study has demonstrated that stakeholders’ knowledge, attitudes and perceptions are critical in effective implementation of disease control measures for both BR and bTB in South Africa. The stakeholders in that specific area of study perceive and believe that the current BR and bTB control measures are not effective. This perception is based on their lived experiences, as they in particular believe the government does not have the resources, operational structure and political drive to implement successful BR and bTB control in the area. One has to be aware that the stakeholders were non-randomly selected and thus their responses may have been influenced by their personal experiences associated with the control of BR or bTB in infected herds in their villages. However, it is concluded that opinions and perspectives of stakeholders need to be considered when disease control strategies are developed and implemented.

## Recommendations

Veterinary officials in the study area need to be capacitated with authority and resources to enable disease control enforcement. Adequate funds need to be allocated for the procurement of the necessary equipment and for human resources to do testing and surveillance. The government should consider implementing a stricter movement control system for all livestock movement (cattle, sheep and goats) in the area, which includes an identification/traceability system and health certification (permits) prior to movement or sale of animals. Vaccination should be made compulsory for BR given the recent outbreaks experienced in the area. Since farmers are agreeable to the testing of their animals, this creates a win–win situation for both farmers and government. Communal infrastructure needs to be upgraded to enable effective separation of livestock from game reserves.

A comprehensive compensation system for livestock owners who forfeit their animals through disease control interventions needs to be put in place. Testing of animals must always be followed-up with report back to farmers and be accompanied by clear decisions on what steps need to be taken with the animals. Private veterinarians in the area have to be roped-in and be authorized to provide some of the veterinary services. Empowerment of farmers with knowledge will ensure that the farmer understands his/her role in disease prevention.

## Limitations

The study was limited to one district in one province in the Republic of South Africa and thus there is a probability of area-specific bias. Wildlife farmers and conservation managers are additional stakeholders and could have added an important perspective.

## Data Availability

Most of the data generated and/or created in the study is contained in the article. Additional data sets are available upon reasonable request.
